# Skeletal Muscle Wasting and Function Impairment in Intensive Care Patients With Severe COVID-19

**DOI:** 10.3389/fphys.2021.640973

**Published:** 2021-03-11

**Authors:** Mario Chueire de Andrade-Junior, Isabel Chateaubriand Diniz de Salles, Christina May Moran de Brito, Laerte Pastore-Junior, Renato Fraga Righetti, Wellington Pereira Yamaguti

**Affiliations:** Hospital Sírio-Libanês, São Paulo, Brazil

**Keywords:** COVID-19, muscle, wasting, function, functionality, ultrasound, SARS-CoV-2 (2019-nCoV)

## Abstract

**Background:** Intensive care patients commonly develop muscle wasting and functional impairment. However, the role of severe COVID-19 in the magnitude of muscle wasting and functionality in the acute critical disease is unknown.

**Objective:** To perform a prospective characterization to evaluate the skeletal muscle mass and functional performance in intensive care patients with severe COVID-19.

**Methods:** Thirty-two critically ill patients (93.8% male; age: 64.1 ± 12.6 years) with the diagnosis of the severe COVID-19 were prospectively recruited within 24 to 72 h following intensive care unit (ICU) admission, from April 2020 to October 2020, at Hospital Sírio-Libanês in Brazil. Patients were recruited if older than 18 years old, diagnosis of severe COVID-19 confirmed by RT-PCR, ICU stay and absence of limb amputation. Muscle wasting was determined through an ultrasound measurement of the rectus femoris cross-sectional area, the thickness of the anterior compartment of the quadriceps muscle (rectus femoris and vastus intermedius), and echogenicity. The peripheral muscle strength was assessed with a handgrip test. The functionality parameter was determined through the ICU mobility scale (IMS) and the International Classification of Functioning, Disability and Health (ICF). All evaluations were performed on days 1 and 10.

**Results:** There were significant reductions in the rectus femoris cross-section area (−30.1% [95% IC, −26.0% to −34.1%]; *P* < 0.05), thickness of the anterior compartment of the quadriceps muscle (−18.6% [95% IC, −14.6% to 22.5%]; *P* < 0.05) and handgrip strength (−22.3% [95% IC, 4.7% to 39.9%]; *P* < 0.05) from days 1 to 10. Patients showed increased mobility (0 [0–5] vs 4.5 [0–8]; *P* < 0.05), improvement in respiratory function (3 [3–3] vs 2 [1–3]; *P* < 0.05) and structure respiratory system (3 [3–3] vs 2 [1–3]; *P* < 0.05), but none of the patients returned to normal levels.

**Conclusion:** In intensive care patients with severe COVID-19, muscle wasting and decreased muscle strength occurred early and rapidly during 10 days of ICU stay with improved mobility and respiratory functions, although they remained below normal levels. These findings may provide insights into skeletal muscle wasting and function in patients with severe COVID-19.

## Introduction

The current outbreak of coronavirus disease 2019 (COVID-19) caused by the severe acute respiratory syndrome coronavirus 2 (SARS-CoV-2) originated in the Hubei Province of the People’s Republic of China (World Health Organization [WHO], 2020). Three months after the emergence, the World Health Organization declared it a pandemic (World Health Organization [WHO], 2020), and several hospitals have produced guidelines for respiratory management of these patients (Lazzeri et al., 2020; Righetti et al., 2020). Studies suggested that up to 12% of hospitalized patients with COVID-19 have required intensive care unit (ICU) hospitalization (Docherty et al., 2020; Richardson et al., 2020).

Survivors among critically ill ICU patients commonly develop severe muscle wasting and impaired muscle function (Iwashyna et al., 2010; Herridge et al., 2011, 2016). Muscle loss may cause impairment of respiratory muscle strength, delaying of weaning from mechanical ventilation, prolonged ICU and hospital stay associated with reduced functional status, and eventually lead to a loss of independence and quality of life (Batt et al., 2017). However, the acute effect of severe COVID-19 in muscle loss and functional impairment is unknown. We thus sought to prospectively characterize and evaluate the time course and magnitude of acute muscle loss in critical illness and determine the role of those alterations in the functional capacity.

## Materials and Methods

### Institutional Context

The present study was conducted at a private tertiary hospital. In period of the study there were 479 beds, of which 327 were in the non-critical units and 152 were in critical care units. Patients with respiratory distress, acute respiratory failure caused by COVID-19 and candidates for respiratory ventilation support were admitted in the critical care units with SARS-CoV-2 dedicated areas. The intubation and invasive mechanical ventilation are indicated for patients with a need for FiO_2_ > 70%, tidal volume (TV) ≥ 9 mL/kg, dependence on ventilatory support (tolerates <2 h without non-invasive ventilation or high-flow nasal cannula) and organ dysfunction. All patients are monitored by the multidisciplinary team that inlcude physicians, nurse, nutritionist, and physiotherapists. In addition, the hospital has an assistance guideline for early mobilization in critical patients for the team of physiotherapists.

### Study Design

All participants included in the study signed the Informed Consent Term, previously approved by the Ethics Committee of the Hospital Sírio-Libanês (number 4.035.714). Patient recruitment was carried out at Hospital Sírio-Libanês, São Paulo, Brazil from April 2020 to October 2020. Inclusion criteria were older than 18 years, SARS-CoV-2 infection confirmed by RT-PCR, ICU stay and the absence of lower limb amputation. The following exclusion criteria were considered: the inability to perform the evaluations within the criteria of technical acceptability and cardiorespiratory instability during the tests (intense dyspnea, angina, presence of arrhythmias, the elevation of the heart rate above 70% of the maximal heart rate predicted by age, and oxygen pulse saturation below 88%). During ICU admission the baseline characteristics of patients and the classification of physical activity before hospitalization were collected. Muscle mass, peripheral muscle strength, mobility, and classification of functioning were performed on days 1 and 10. Patients were followed up from admission to the ICU, therefore, on the 10th day evaluation, patients could be in other hospital units dedicated to assisting patients with COVID-19.

### Level of Physical Activity

The prehospitalization physical activity level was assessed by the International Physical Activity Questionnaire-Short Form (IPAQ-SF) based on Craig et al. (2003) and Matsudo et al. (2001). The questionnaire includes questions about time spent sitting as an indicator of sedentary behavior. In each of the four domains, the number of days per week and time per day spent in both moderate and vigorous activity were registered. The patients were classified into five categories according to the frequency and duration of the activities evaluated (highly active, active, irregularly active A, irregularly active B, and sedentary), accordingly to International Physical Activity Questionnaire Committee (International Physical Activity Questionnaire Committee, 2005).

### Severity of COVID-19

The severity of COVID-19 was defined according to the guidelines for COVID-19 issued by the National Institutes of Health (2020) and classified as Asymptomatic infection (positive test for SARS-CoV-2 but the patients have no symptoms); mild disease (show various signs and symptoms of fever, cough, sore throat, malaise, headache, muscle pain without shortness of breath, dyspnea, or abnormal chest imaging); moderate disease (evidence of lower respiratory disease by clinical assessment or imaging and SpO_2_ ≥ 94% on room air at sea level); severe disease (respiratory frequency >30 breaths per minute, SpO_2_ < 94% on room air at sea level, a ratio of the arterial partial pressure of oxygen to fraction of inspired oxygen (PaO_2_/FiO_2_) < 300 mmHg or lung infiltrates >50%); and critical disease (develop respiratory failure, septic shock, and/or multiple organ dysfunction).

### Measurement of Muscle Mass and Echogenicity

Muscle mass loss was assessed by means of ultrasonography. Rectus femoris cross-sectional area (cm^2^) and the thickness of the anterior compartment of the quadriceps muscle (rectus femoris and vastus intermedius) (cm) were measured using B-mode ultrasound (Logiq e ultrasound, GE Healthcare, United States) (Seymour et al., 2009). Echogenicity was determined by gray-scale analysis using Image J (National Institutes of Health, United States). The depth setting was fixed at 6 cm and the rectus femoris muscle region was selected to include as much of the muscle as possible, excluding bone or surrounding fascia. The mean echogenicity of the rectus femoris muscle region was calculated with an 8-bit resolution, resulting in a number between 0 and 255 (black = 0 and white = 255) and averaged over the three measurements (Arts et al., 2010). The results were expressed considering the average of three measurements.

### Peripheral Muscle Strength Assessment

A manual hydraulic dynamometer (Jamar Hydraulic Hand Dynamometer (Sammons Preston Rolyan, Chicago, IL, United States) was used for peripheral muscle strength assessment. The recommendations for the handgrip strength test of the American Society of Hand Therapists were followed (Fess, 1992). The patient was in sitting position and the non-dominant hand resting on the thigh. Regarding the evaluated dominant upper limb, the patient was instructed to keep the shoulder in a neutral position, elbow in 90° flexion, and the forearm in neutral rotation. The patients were instructed to grip the dynamometer with maximum strength in response to a standardized voice command (Lopes et al., 2018). The rest period between measurements was at least 1 min. Three measurements were taken, considering a difference lower than or equal to 10%. The highest value was considered for analysis (Fess, 1992; Lopes et al., 2018; Francisco et al., 2020).

### Mobility in the ICU

Intensive care mobility was assessed by the ICU Mobility Scale (IMS). IMS has scores ranging from 0 to 10 according to the mobility activities. The IMS allows scoring mobility of the patients from the moment in which they are bed-ridden until they are walking independently. The scale considers the need for assistance from another person or walking device (Hodgson C. et al., 2014). Higher scores in the IMS are associated with greater mobility (Hodgson et al., 2013). The IMS has strong interrater reliability (Hodgson C. et al., 2014), validity (Parry et al., 2015), construct validity with muscle strength (Tipping et al., 2016), and its translation and cultural adaptation have been carried out in Portuguese (Kawaguchi et al., 2016).

### International Classification of Functioning, Disability, and Health

The functionality was assessed by the International Classification of Functioning, Disability, and Health (ICF) based on Paschoal et al. (2019). The ICF domains used were muscle strength (b730), respiratory function (b440), structure of the respiratory system (s430), and walking (d450) scored according to Table 1.

**TABLE 1:** The ICF domains for muscle strength, respiratory function, structure of the respiratory system, and walking.

**ICF Domains and score**
**b730 – Muscle strength**
**MRC score for global strength**
0 – 58 to 60 (without significant changes)
1 – 48 to 57 (slight loss)
2 – 31 to 47 (moderate loss)
3 – 4 to 30 (severe loss)
4 – 0 to 3 (maximum loss)

**b440 – Respiratory function**
0 – SpO_2_ ≥ 90% or ≥ 88% in chronic pulmonary disease in room air
1 – Oxygen therapy with ≤ 2 L/minO_2_ or FiO_2_ < 28% for saturation target (90–93%), requires physiotherapy, but not ventilatory support
2 – Requires ventilatory support in a shorter period of the time or use of 2 to 5 L/minO_2_ or FiO_2_ 28 to 40%.
3 – Requires ventilatory support most of the time or use of 5.1 to 9 L/minO_2_ or FiO_2_ > 40 to 99%
4 – Requires ventilatory support all the time or use of ≥10 L/minO_2_ or FiO_2_ = 100%

**s430 – Structure of the respiratory system (pulmonary involvement)**
0 – no changes
1 – Mild involvement (reduction of expansibility or laminar atelectasis)
2 – Moderate alteration (requiring exercise with non-invasive ventilation or moderate banded atelectasis)
3 – Severe alteration (uncomplicated bronchopneumonia or atelectasis of a lobe, with a greater need for non-invasive ventilation)
4 – Total involvement (complicated bronchopneumonia, atelectasis of a lung, and the mechanical ventilation needed)

**d450** – **Walking**
0 – No disability
1 – Mild disability (slight lameness or need to use a walking stick)
2 – Moderate disability (moderate deficit or need to use a walker)
3 – Severe disability (gait with the aid of others or therapeutic gait)
4 – Inability to walk

*MRC, Medical Research Council; SpO_2_, peripheral oxygen saturation; FiO_2_, Fraction of inspired oxygen.*

### Statistical Analysis

Data were assessed for normality using the Shapiro-Wilk test. Parametric variables are presented as mean and non-parametric variables as median and interquartile range. Categorical data are presented as the absolute (*n*) and relative frequency (%) and compared by the χ^2^ test or Fisher’s exact test between patients with and without invasive mechanical ventilation. Data were then analyzed using the *t*-test, Spearman’s rank correlation, Mann–Whitney test, and the Wilcoxon signed-rank test as appropriate. Statistical significance was indicated by a *P*-value of less than.05.

## Results

**FIGURE 1:**
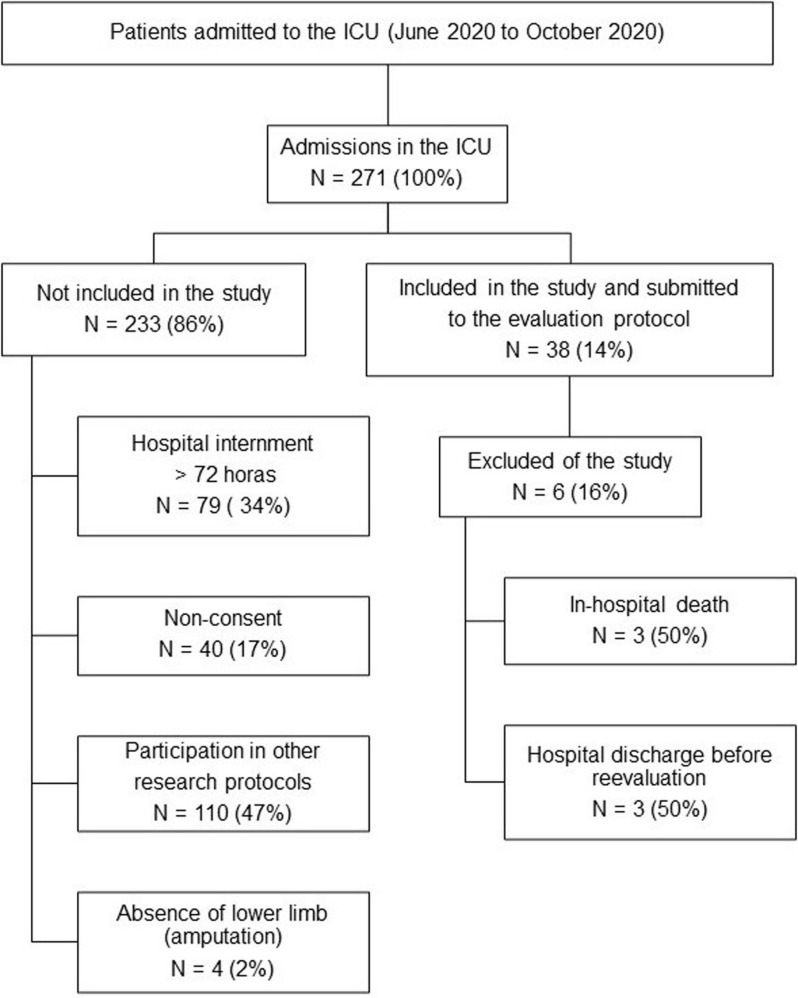
Diagram of the study. ICU, intensive care unit.

From April 20, 2020 to October 20, 2020, a total of 271 patients with confirmed SARS-CoV-2 infection were hospitalized in the ICU. Among these patients, 38 met the inclusion criteria. Six patients were excluded (three deaths and three hospital discharges before day 10). Therefore, 32 patients were analyzed (Figure 1). Table 2 shows the demographic and clinical characteristics of the patients.

**TABLE 2:** Demographic and clinical characteristics of the patients.

	**All patients (*N* = 32)**	**Patients with IMV (*N* = 24)**	**Patients without IMV (*N* = 8)**	***P*-value**
Age, mean (±SD), years	64.1 ± 12.6	66.9 ± 11.6*	55.7 ± 12.4	<0.05
Male sex. No. (%)	30 (93.8)	23 (95.8)	7 (87.5)	N.S.
IMV days, median (range), days	6 [2–9.5]	8.5 [6–10]*	0 [0–0]	<0.05
ICU length of stay, median (range), days	10 [6–10]	10 [9–10]*	6 [5.5–6]	<0.05
Hospital length of stay, median (range), days	21 [15–42]	24.5 [18–56]*	14 [12–16]	<0.05
APACHE II score, median (range)	19.0 [6.5–22]	20.5 [16.5–22.5]*	5.5 [3.0–8.5]	<0.05
SOFA score, median (range)	6 [1–8.5]	7 [6–9.5]*	0.5 [0–1]	<0.05
Smoker, No. (%)	0 (0%)	0 (0%)	0 (0%)	N.S.
BMI (kg/m^2^)	28.3 ± 3.9	28.3 ± 3.8	28.3 ± 4.7	N.S.
**IPAQ-SF before ICU admission, No. (%)**				
Highly active	2 (6.3)	1 (4.2)	1 (12.5)	N.S.
Active	18 (56.3)	12 (50)	6 (75)	N.S.
Irregularly active A	0 (0)	0 (0)	0 (0)	N.S.
Irregularly active B	2 (6.3)	2 (8.3)	0 (0)	N.S.
Sedentary	10 (31.3)	9 (37.5)	1 (12.5)	N.S.
**COVID-19 severity**				
Severe illness	8 (25)	0 (0)	8 (100)	
Critical illness	24 (75)	24 (100)	0 (0)	
**Therapeutic strategies**				
Midazolam median (range), mg	50 [25–50]	50 [50–50]*	0 [0–0]	<0.05
Midazolam median (range), days	4.5 [0.5–10]	6.5 [4–10]*	0 [0–0]	
Fentanyl, median (range), mcg	50 [25–50]	50 [50–50]*	0 [0–0]	<0.05
Fentanyl, median (range), days	4.5 [0.5–10]	6.5 [4–10]*	0 [0–0]	<0.05
Noradrenaline, median (range), mcg	0 [0–6]	0 [0–11]*	0 [0–0]	<0.05
Use of neuromuscular blocking agents, median (range), days	1 [0–5]	3 [0–6]*	0 [0–0]	<0.05
**Hydrocortisone dose, median (range), mg**				
Day 1	80 [80–80]	80 [80–80]	80 [70–80]	N.S.
Total by day 10	660 [500–735]	690 [580–800]*	495 [390–600]	<0.05
High-flow nasal cannula oxygen therapy, No. (%)	16 (50)	8 (33.3)	8 (100)	N.S.
Dialysis, No. (%)	1 (3.1)	1 (4.2)	0 (0)	N.S.
ECMO, No. (%)	2 (6.3)	2 (8.3)	0 (0)	N.S.
**Laboratory findings**				
Blood glucose level, median (range), mg/dL	178.5 [141.0–208.0]	182 [157.5–207.5]	137 [106.5–222.5]	N.S.
C-reactive protein, (±SD), mg/dL				
Day 1	12.5 ± 7.6	12.5 ± 8.4	12.5 ± 5.3	N.S.
Day 10	1.7 ± 2.0	2.2 ± 2.1*	0.3 ± 0.09	<0.05
D-dimer, median (range), ng/mL	1047.5 [719–1650]	1133.5 [812–1819.5]	680 [501–1190]	N.S.
**Comorbidities, No. (%)**				
Hypertension	21 (65.6)	16 (66.7)	5 (62.5)	N.S.
Diabetes Mellitus	10 (31.3)	8 (33.3)	2 (25)	N.S.
Obesity	8 (25)	6 (25)	2 (25)	N.S.
Dyslipidemia	6 (18.8)	4 (16.7)	2 (25)	N.S.
Anxiety	3 (9.4)	1 (4.2)	2 (25)	N.S.
Depression	2 (6.3)	1 (4.2)	1 (12.5)	N.S.
Obstructive sleep apnea syndrome	4 (12.5)	3 (12.5)	1 (12.5)	N.S.
Acute myocardial infarction	2 (6.3)	2 (8.3)	0 (0)	N.S.
**Nutritional information**				
Caloric intake goals, median (range), Kcal/day				
Day 1	1610.0 ± 257.1	1603.6 ± 279.3^#^	1629.3 ± 189.3	N.S.
Day 10	1846.6 ± 222.6	1836.0 ± 203.0	1878,3 ± 287.3	N.S.
Protein intake goals, median (range), g/day				
Day 1	96.6 ± 13.3	97.9 ± 13.5^#^	92.6 ± 12.6	N.S.
Day 10	99.0 ± 15.7	101.1 ± 15.8	92.8 ± 14.3	N.S.

BMI, body mass index. *P < 0.05 vs patients without IMV. ^#^*P* < 0.05 vs Day 10.

### Changes in Muscle Mass and Echogenicity

**FIGURE 2:**
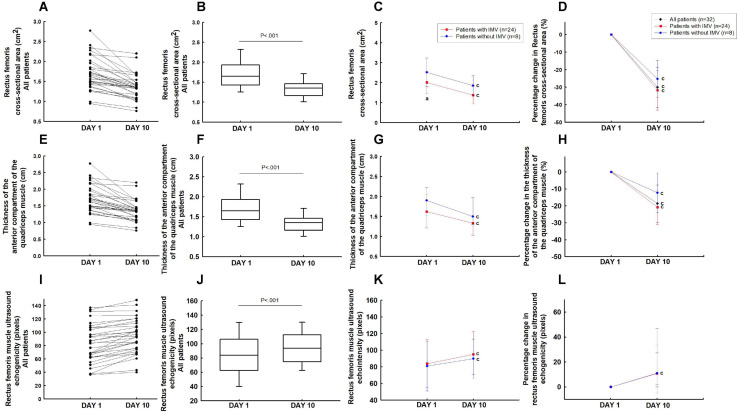
Ultrasound muscle assesses the rectus femoris cross-section area **(A–D)**, the thickness of the anterior compartment of the quadriceps muscle (rectus femoris and vastus intermedius) **(E–H)**, and rectus femorisechogenicity **(I–L)**. ^a^*P* < 0.05 vs patients without IMV on day 1; ^c^*P* < 0.05 vs day 1.

In the overall group, the rectus femoris cross-sectional area (−30.1% [95% IC, −26.0 to −34.1%]; *P* < 0.001) and the thickness of the anterior compartment of the quadriceps muscle (−18.6% [95% IC, −14.6 to 22.5%]; *P* < 0.001) decreased significantly from days 1 to 10 (Figures 2A,B,D–F,H). These patients showed a reduction of 3.7% [95% IC, 3.2 to 4.2%] per day in the rectus femoris cross-sectional area and 2.1% [95% IC, 1.5 to 2.6%] per day in the thickness of the anterior compartment of the quadriceps muscle during the ICU stay.

Patients that required IMV showed lower rectus femoris cross-sectional area on day 1 compared to the patients without IMV (*P* < 0.05) (Figure 2C). The echogenicity increased from days 1 to 10 in the overall group (16.8% [95% IC, 22.9 to 10.7%]; *P* < 0.05) (Figures 2I,J). There was no difference in the rectus femoris cross-sectional area (Figures 2C,D), the thickness of the anterior compartment of the quadriceps muscle (Figures 2G,H), and echogenicity (Figures 2K,L) between patients with and without IMV on day 10.

**FIGURE 3:**
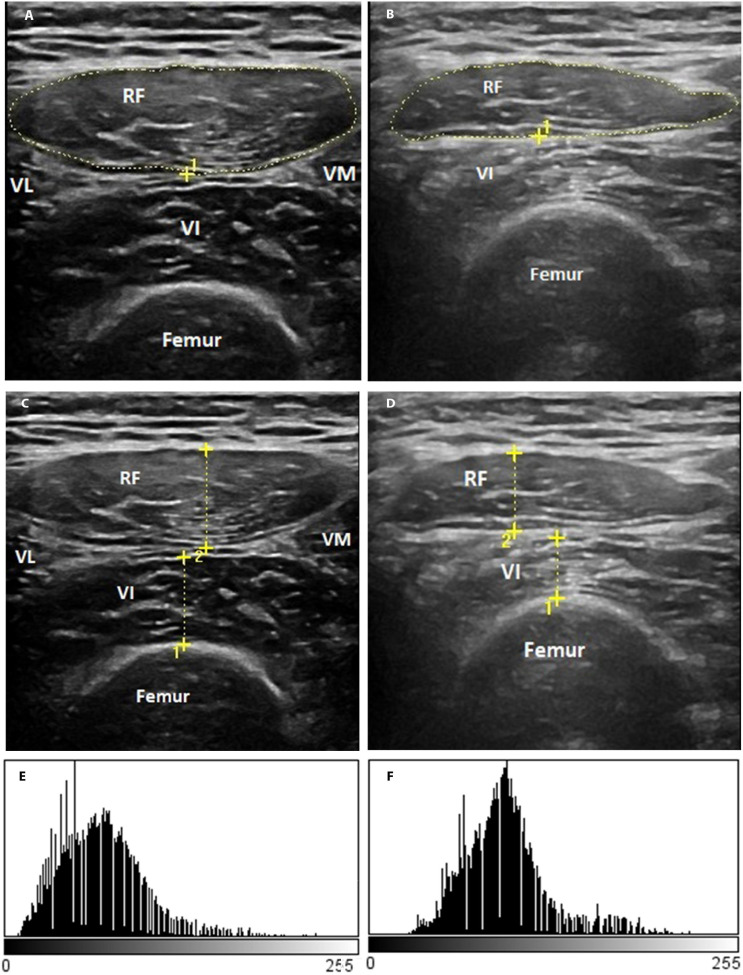
Representative qualitative muscle ultrasound image: rectus femoris cross-section area **(A,B)**, the thickness of the anterior compartment of the quadriceps muscle (rectus femoris and vastus intermedius) **(C,D)**, and rectus femoris echogenicity **(E,F)**. The representative image is of a male patient, a 55-year-old man with a positive diagnosis for SARS-CoV-2 infection and that showed a severe illness.

Figure 3 shows the representative rectus femoris cross-sectional area (Figures 3A,B), the thickness of the anterior compartment of the quadriceps muscle (Figures 3C,D) ultrasound images, and echogenicity (Figures 3E,F) of a patient with severe COVID-19 from days 1 to 10.

### Changes in Peripheral Muscle Strength

**FIGURE 4:**
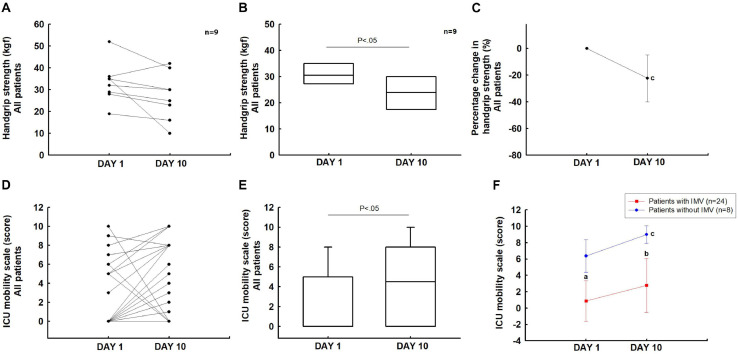
Muscle strength and mobility: handgrip strength **(A–C)** and IMS scale **(D–F)**. ^a^*P* < 0.05 vs patients without IMV on day 1; ^b^*P* < 0.05 vs patients without IMV on day 10; ^c^*P* < 0.05 vs day 1.

Twenty-three patients did not complete this assessment because they were sedated and on IMV. Therefore, we evaluated nine patients (one with IMV and eight without IMV) who completed the handgrip test on days 1 and 10. In these patients, the handgrip strength decreased significantly from days 1 to 10 (*P* < 0.05). On day 10, there was a reduction of 22.3% ([95% IC, 4.7 to 39.9%]; *P* < 0.05) compared to the baseline (Figures 4A–C).

### Changes in Mobility

In the overall group, the IMS score increased significantly from days 1 to 10 (0 [0–5] vs 4.5 [0–8]; *P* < 0.05) (Figures 4D,E). Patients without IMV showed an increase in the IMS score from days 1 to 10 (0 [0–0] vs 1 [0–5.5]; *P* < 0.05). The group with IMV showed a significant difference on day 1 (0 [0–0] vs 6.5 [5–8]; *P* < 0.05) and day 10 when compared to patients without IMV (1 [0–5.5] vs 9 [8–10]; *P* < 0.05) (Figure 4F).

### Changes in the Classification of Functioning

**FIGURE 5:**
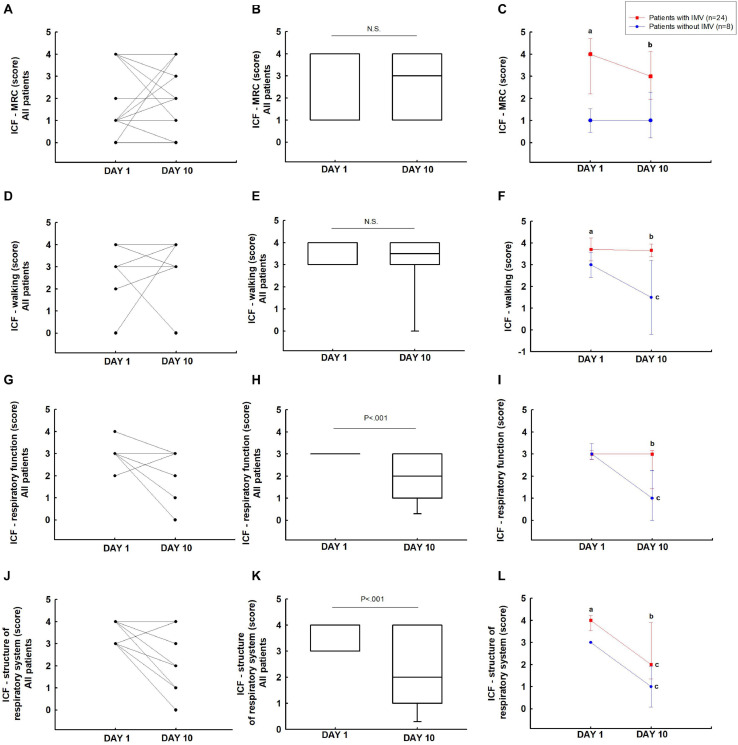
Function assessed: ICF-MRC **(A–C)**, ICF-walking **(D–F)**, ICF-respiratory function **(G–I)**, and ICF-structure respiratory system **(J–L)**. ^a^*P* < 0.05 vs patients without IMV on day 1; ^b^*P* < 0.05 patients without IMV on day 10; ^c^*P* < 0.05 vs day 1.

In the ICF-MRC (Figures 5A,B) and ICF-walking score (Figures 5D,E), the overall patients did not show difference from days 1 to 10. The patients with IMV showed a significant difference in the ICF-MRC (4 [4–4] vs 1 [1–1]; *P* < 0.05) and ICF-walking (4 [4–4] vs 3 [3–3]; *P* < 0.05) scores on day 1 compared to patients without IMV. It was also observed on day 10 (ICF-MRC: 3 [3–4] vs 1 [0.5–2] and ICF-walking: 4 [4–4] vs 1.5 [0–3]; *P* < 0.05) (Figures 5C,F).

International Classification of Functioning, Disability and Health-respiratory function in the overall group showed an improvement in respiratory function from days 1 to 10 (3 [3–3] vs 2 [1–3]; *P* < 0.05) (Figures 5G,H). Patients without IMV showed improvement in respiratory function on day 10 (3 [3–3] vs 1 [0–2]; *P* < 0.05). The patients with IMV showed a significant difference when compared to patients without IMV on day 10 (3 [1.5–3] vs 1 [0–2]; *P* < 0.05) (Figure 5I).

ICF-structure of the respiratory system in the overall group showed an improvement in the ICF-structure of the respiratory system from days 1 to 10 (4 [3–4] vs 2 [1–4]; *P* < 0.05) (Figures 5J,K). In patients with IMV (4 [4–4] vs 2 [1.5–4]; *P* < 0.05) and without IMV (3 [3–3] vs 1 [0–2]; *P* < 0.05) there was an improvement in the ICF-structure of the respiratory system from days 1 to 10 (*P* < 0.05). The patients with IMV showed a significant difference when compared to patients without IMV on days 1 (4 [4–4] vs 3 [3–3]; *P* < 0.05) and 10 (2 [1.5–4] vs 1 [0–2]; *P* < 0.05) (Figure 5L).

### Correlation Between ICU Length of Stay, Hydrocortisone Dose, Rectus Femoris Cross-Sectional Area, Thickness of the Anterior Compartment of the Quadriceps Muscle, and Handgrip Strength, Age, Mobility, and ICF Domains

Spearmans’s correlations show a very strong correlation of handgrip strength with IMS score (*r* = 0.98, *P* = 0.0000002), ICF-MRC (*r* = −0.97, *P* = 0.0000002), ICF-respiratory function (*r* = −0.85, *P* = 0.0000002) and ICF-structure of respiratory system score (*r* = −0.89, *P* = 0.0000002); a moderate correlation of the rectus femoris cross-sectional area with age (*r* = −0.62, *P* = 0.0001) and fair correlation with ICF-walking (*r* = −0.37, *P* = 0.032). There was no correlation between the rectus femoris cross-sectional area and thickness of the anterior compartment of the quadriceps muscle with handgrip strength.

The correlations show a very strong correlation of ICU length stay with handgrip strength (*r* = −0.84, *P* = 0.0000002), IMS score (*r* = −0.84, *P* = 0.0000002), ICF-MRC (*r* = 0.84, *P* = 0.0000002) and ICF-structure of respiratory system score (*r* = 0.73, *P* = 0.0000002) and moderate correlation of ICU length stay with ICF-respiratory function (*r* = 0.67, *P* = 0.00001). There was no correlation between the ICU length stay with rectus femoris cross-sectional area, thickness of the anterior compartment of the quadriceps muscle, echogenicity.

Concerning hydrocortisone, the correlations show a weak correlation of hydrocortisone dose with handgrip strength (*r* = −0.49, *P* = 0.003) and ICF-MRC (*r* = 0.50, *P* = 0.003) and moderate correlation of hydrocortisone dose with ICF-walking (*r* = 0.60, *P* = 0.003). There was no correlation between the hydrocortisone dose with rectus femoris cross-sectional area, thickness of the anterior compartment of the quadriceps muscle, and echogenicity.

## Discussion

Severe and critical COVID-19 patients showed a reduction in cross-sectional rectus femoris area and the thickness of the anterior compartment of the quadriceps muscle (rectus femoris and vastus intermedius) with increased echogenicity from days 1 to 10. Furthermore, these patients also showed a reduction in handgrip strength. Muscle wasting was similar in patients with and without IMV. However, patients showed an increase in the mobility scale on day 10 and improvement in the ICF-respiratory function and ICF-structure of respiratory system scores.

In the present study, patients with COVID-19 had a reduction of 30.1% in the rectus femoris cross-sectional area and 18.6% in the thickness of the anterior compartment of the quadriceps muscle on day 10. The magnitude of these reductions was about 3.7 and 2.1% per day, respectively. Puthucheary et al. (2013) showed that muscle wasting occurred early and rapidly during the first week of critical illness with a reduction of 17.7% in the rectus femoris muscle area on day 10. In the same study, patients with failure of four organs showed muscle loss of more than approximately 27% by the end of ten days. However, in our study, patients with COVID-19 without a multiorgan failure affecting more than three organs showed greater muscle weight loss compared to the study by Puthucheary et al. (2013).

Hayes et al. (2018) showed a reduction in the quadriceps muscle thickness in critically ill patients with extracorporeal membrane oxygenation, but there was no change in the echogenicity of the femoral rectus muscle on day 10. In the current study, the loss in the thickness of the anterior compartment of the quadriceps muscle also occurs in patients with COVID-19. However, these patients showed an increase in rectus femoris echogenicity. Such observations suggest deterioration in muscle quality (Formenti et al., 2019). It could be suggested that the increase in rectus femoris echogenicity may be representative of the infiltration of fatty and connective tissue and muscle necrosis associated with the remodeling of muscle fibers. Grimm et al. (2013) showed that echogenicity score increases from days 4 to 14 in the presence of decreasing fluid balances on day 14. This is supported by findings from muscle magnetic resonance imaging in a patient on day 14 which showed significant changes in muscle architecture without tissue edema, confirming a specific structural damage in muscle architecture. Several studies, albeit not including the COVID-19 nor ICU patients, have demonstrated a correlation between echogenicity and measurements of fibrosis and intramuscular fat (Reimers C. D. et al., 1993; Reimers K. et al., 1993).

Patients showed reduction in handgrip strength on day 10. Known risk factors for ICU-acquired weakness include older age, diagnosis of inflammatory disease, electrolyte disturbances, receipt of corticosteroids and neuromuscular blocking agents, illness severity, and immobility (Schweickert et al., 2009; Chlan et al., 2015). Furthermore, handgrip strength is considered an indicator of global muscle function and is also independently associated with poor hospital outcomes in critical illness (Ali et al., 2008; Porto et al., 2019).

Inflammation reduces muscle protein synthesis and increases muscle degradation (Vesali et al., 2010). In clinical and experimental studies it has been shown that lung-derived inflammatory mediators are associated with muscle wasting in acute and chronic inflammatory lung disease (de Godoy et al., 1996; Files et al., 2016). Currently, no studies have shown acute muscle wasting in patients with critical COVID-19. However, studies showed that these patients with COVID-19 present a high expression of tumor necrosis factor (TNF)-α, interleukin (IL)-1β, and IL-6 (Huang et al., 2020; Tang et al., 2020). These cytokines can modulate muscle protein synthesis (Cohen et al., 2015; Zhou et al., 2016).

The balance of muscle protein synthesis and breakdown is modulated by the anabolic and catabolic signals. Inflammation is not only a marker of severe disease but also a powerful driver of muscle catabolism and atrophy via cytokine-mediated activation of the ubiquitin-proteasome and lysosomal hydrolases system necessary for proteolysis (Bechet et al., 2005; Schiaffino et al., 2013). The muscle inflammation, evidenced by inflammatory infiltrates of leukocytes in muscle biopsies of patients with critical illness is often concurrent with muscle hypoxia and both are closely related to reduces protein synthesis and the anabolic process is commonly shown in the early phase of critical illness (Dos Santos et al., 2016; Puthucheary et al., 2018).

Amino acids and other nutrients are an important substrate to build muscle protein and muscle maintenance. Also, these substrates serve as regulators of muscle protein synthesis on the cell. The increase of amino acids in the intracellular activates the mammalian target of rapamycin (mTOR), which is the main pathway driving protein synthesis (Palm and Thompson, 2017). Corroborating with the hypothesis, Arunachalam et al. (2020) published a recent study with 76 COVID-19 patients that showed a reduced expression of human leukocyte antigen (HLA)-DR and pro-inflammatory cytokines in myeloid cells, and impaired mTOR-signaling. Furthermore, patients with critical illness showed the endogenous nutrients are diverted away from the muscle as muscle-bound amino acids are released into the systemic circulation, explained to be an adaptive response to meet the increased systemic metabolic demands Preiser et al. (2014). In the present study, the needs for caloric and protein intake were individually planned for each patient and according to the clinical need. Corroborating that the results exclude factors of lack of supplementation of calories or exogenous proteins.

In the current study mobility level improved from days 1 to 10 (although it remained below normal levels) despite a reduction in rectus femoris cross-sectional area, the thickness of the anterior compartment of the quadriceps muscle, and handgrip strength over this period. Mobility level in the ICU depends not only on the function, mass, or quality of the muscles but also on the neural adaptation and cardiorespiratory functional reserve, which was especially impaired in the first days of the ICU admission (Sale, 1988; Hodgson C. L. et al., 2014; McGregor et al., 2014). The improvement in the mobility level was accompanied by the reestablishment of the respiratory function over time. Patients were also more likely to be off IMV from days 1 to 10, and not requiring sedation. This resulted in patients participating in higher levels of physiotherapy, including ambulation.

The patients showed improvement in the ICF-respiratory function and ICF-structure of respiratory system scores. This improvement can be explained by the clinical course of the COVID-19 and increased mobility of these patients. Corroborating our results, Schujmann et al. (2018), showed that mobility and exercise improve respiratory function in critically ill patients, besides the favorable clinical course. It is important to note that the ICU where the data were collected in the current study presents a structured early mobilization guideline (Murakami et al., 2015). In addition, COVID-19 surviving patients have an average ICU stay of 13 days, showing that in our study the patient was also in clinical improvement of the disease (Zhou et al., 2020).

Muscle loss was similar in patients with and without IMV. The mechanisms of loss of muscle mass and strength are previously well known (Schweickert et al., 2009; Chlan et al., 2015). However, the magnitude of the loss of muscle strength and functional capacity in patients without IMV are not totally understood. Suesada et al. (2007), showed that short-term hospitalization reduces functional capacity in non-critical patients, regardless of age or initial functional status. Therefore, mobility restriction is probably a determining factor in these patients.

This study has some limitations. This was a single-center study, and the findings may not be generalizable to other settings. However, the single-center setting also might be seen as a strength because the sedation, ventilator weaning, and early mobilization protocols were similar in both groups, but a reduced number of individuals composing the sample could influence the extrapolation of the findings to other hospitals. Ultrasound measurement can be influenced by observer-dependent factors, such as the adjustment of the ultrasound probe. However, our study maintained a single trained evaluator to assess ultrasound muscle mass.

The present study is the first study to report muscle mass loss in patients with COVID-19. However, the lack of a control group limits the comparison with other critical illnesses. Despite this, the objective of this study was to document this loss in this specific population with COVID-19, which occurs in a similar way to other critical diseases. In addition, our study shows these changes in patients without invasive mechanical ventilation. Additionally, factors, such as hydration balance, might also have an impact on image analysis.

Patients with IMV showed differences in age, length of stay in the ICU, hidrocortisone total by day 10, fentanyl, midazolam and noradrenaline dose when compared to the group without IMV. A recent meta-analysis of 33 studies conducted on a total of 3,027 patients with COVID-19 showed that adults over 65 years old were five times more likely to become a critical illness or die Zheng et al. (2020). Therefore, patient characteristics, such as age and comorbidities, affect the severity of the disease and are likely to influence the length of stay (Clark et al., 2020; Rodriguez-Morales et al., 2020; Wu and McGoogan, 2020). In addition, the World Health Organization guideline on drugs for COVID-19 suggests the use of corticosteroids in severe and critical cases (Siemieniuk et al., 2020). The most critical presentation of the disease may have directed the physicians team to treat the IMV group with corticosteroids. Finally, the current study shows muscle loss and functionality parameters in the acute and critical phase of patients with COVID-19. However, additional studies are needed to identify long-term residual changes in muscle mass and functionality parameters.

In conclusion, severe and critically ill patients with COVID-19, muscle wasting, and decreased muscle strength occurred early and rapidly during 10 days of ICU stay with improved mobility and respiratory functions, although they remained below normal levels. These findings may provide insights into skeletal muscle wasting and functionality in patients with COVID-19.

## Data Availability Statement

The original contributions presented in the study are included in the article, further inquiries can bedirected to the corresponding author/s.

## Ethics Statement

The studies involving human participants were reviewed and approved by the Ethics Committee of the Hospital Sírio-Libanês (number 4.035.714). The patients/participants provided their written informed consent to participate in this study.

## Author Contributions

MA-J, IS, CB, RR, and WY: concept and design. MA-J, IS, RR, and WY: acquisition, analysis, or interpretation of data. MA-J, RR, and WY: drafting of the manuscript. MA-J, IS, CB, LP-J, RR, and WY: critical revision of the manuscript for important intellectual content. RR and WY: statistical analysis. LP-J and WY: administrative, technical, or material support. WY: supervision. All authors contributed to the article and approved the submitted version.

## Conflict of Interest

The authors declare that the research was conducted in the absence of any commercial or financial relationships that could be construed as a potential conflict of interest.
